# Enhancing Teak (*Tectona grandis*) Seedling Growth by Rhizosphere Microbes: A Sustainable Way to Optimize Agroforestry

**DOI:** 10.3390/microorganisms9091990

**Published:** 2021-09-19

**Authors:** Leardwiriyakool Chaiya, Paiboolya Gavinlertvatana, Neung Teaumroong, Wasu Pathom-aree, Amornrat Chaiyasen, Rungroch Sungthong, Saisamorn Lumyong

**Affiliations:** 1 Department of Biology, Faculty of Science, Chiang Mai University, Chiang Mai 50200, Thailand; Leardkool@gmail.com (L.C.); wasu.p@cmu.ac.th (W.P.-a.); 2 Thai Orchid Labs Co., Ltd., Khannayao, Bangkok 10230, Thailand; thaiorchidslab@gmail.com; 3 School of Biotechnology, Institute of Agricultural Technology, Suranaree University of Technology, Nakhon Ratchasima 30000, Thailand; neung@sut.ac.th; 4 Center of Excellence in Microbial Diversity and Sustainable Utilization, Faculty of Science, Chiang Mai University, Chiang Mai 50200, Thailand; 5 Soil Science Research Group, Agricultural Production Science Research and Development Division, Department of Agriculture, Ministry of Agriculture and Cooperatives, Bangkok 10900, Thailand; amornrat057@gmail.com; 6 Laboratory of Hydrology and Geochemistry of Strasbourg, University of Strasbourg, UMR 7517 CNRS/EOST, 67084 Strasbourg, France; 7 Academy of Science, The Royal Society of Thailand, Bangkok 10300, Thailand

**Keywords:** actinobacteria, agroforestry, arbuscular mycorrhizal fungi, beneficial microbes, nitrogen-fixing bacteria, plant-microbe interactions, teak

## Abstract

With its premium wood quality and resistance to pests, teak is a valuable tree species remarkably required for timber trading and agroforestry. The nursery stage of teak plantation needs critical care to warrant its long-term productivity. This study aimed to search for beneficial teak rhizosphere microbes and assess their teak-growth-promoting potentials during nursery stock preparation. Three teak rhizosphere/root-associated microbes, including two teak rhizobacteria (a nitrogen-fixing teak root endophyte-*Agrobacterium* sp. CGC-5 and a teak rhizosphere actinobacterium-*Kitasatospora* sp. TCM1-050) and an arbuscular mycorrhizal fungus (*Claroideoglomus* sp. PBT03), were isolated and used in this study. Both teak rhizobacteria could produce in vitro phytohormones (auxins) and catalase. With the pot-scale assessments, applying these rhizosphere microbes in the form of consortia offered better teak-growth-promoting activities than the individual applications, supported by significantly increased teak seedling biomass. Moreover, teak-growth-promoting roles of the arbuscular mycorrhizal fungus were highly dependent upon the support by other teak rhizobacteria. Based on our findings, establishing the synergistic interactions between beneficial rhizosphere microbes and teak roots was a promising sustainable strategy to enhance teak growth and development at the nursery stage and reduce chemical inputs in agroforestry.

## 1. Introduction

Teak (*Tectona grandis*)—a sizeable deciduous dicot tree that belongs to the family *Verbenaceae*—is an indigenous plant in South and Southeast Asia’s tropical and subtropical zones [1]. Over 29,000 million hectares of natural teak forest are in India, Laos, Myanmar, and Thailand [2]. The teak plantation area is around 4346 million hectares and accounts for a total of 38 countries, in Asia (83%), Africa (11%), and tropical America (6%) [2]. Thailand is among the top ten countries, holding the largest planting area of teak. In 2010, the United Nations’ Food and Agriculture Organization reported that Thailand’s natural teak forest and teak plantation covered 8,744,000 and 128,000 hectares, respectively [2]. Teak is considered one of the world’s most valuable trees, as it is a source of premium hardwoods for diverse applications (e.g., furniture, construction, panel work, railway carriages) [3]. With its excellent quality and commercial values, teak is often a popular hardwood in the wood industry and timber trading [4]. Besides, this tree species is well-known for its durability to withstand weather conditions and pests, making it a popular plant for reforestation and agroforestry [5,6].

Although teak is a pest-resistant plant and its microbial diseases are relatively rare, some microbes have been reported occasionally as causative agents of various teak diseases, such as wilt disease caused by *Ceratocystis fimbriata* [7], brown leaf spot disease caused by *Alternaria alternata* [8], crown gall disease caused by *Agrobacterium tumefaciens* species complex [9], and root collar rot disease caused by *Kretzschmaria zonata* [10]. These emerging diseases of teak often occur during the nursery stage (the most critical step for long-lived teak), where inappropriate growth conditions, such as insufficient drainage, high humidity, and soil-borne pathogenic infestation are inducing factors. The increase of teak disease incidence is a long-term threat to teak health and quality, which can further cause severe loss and outbreak in the wood industry and forestry. Some synthesized pesticides and fertilizers have been used to prepare teak seedlings, aiming to maintain the teak’s survival rate at the early growth phase (~2 years) and ensure maximum tree development in the following years [11,12,13]. However, applying agrochemicals in teak seedling preparation may endanger beneficial teak-associated microbes and agroforestry health (e.g., pesticide bioaccumulation into the ecological food web, increasing resistance development in forest phytopathogens).

An alternative approach to supporting sustainable growth and development of teak is to utilize beneficial teak-associated microbes. It is conceivable that many rhizosphere microbes provide various benefits to their commensal plants, such as facilitating soil nutrients/minerals uptake of plants, producing essential plant hormones, optimizing plant defensive systems against phytopathogens [14,15,16]. Some selected microbial consortia made of plant growth-promoting rhizobacteria (e.g., *Azotobacter chroococcum*, *Bacillus subtilis*) and arbuscular mycorrhizal fungi (e.g., *Ambispora leptoticha*, *Rhizophagus clarus*) have proved their ability to enhance nursery growth and development of teak either under greenhouse conditions or field trials [16,17]. To our knowledge, teak rhizosphere microbial community structure and how different teak-associated rhizosphere microbes work together in promoting teak growth and development is still largely unknown. A study revealed that converting agroforestry practices from diverse native forests to monoculture (teak) plantations affected microbial communities and forest soil functioning [18]. Interestingly, the abundance of bacterial taxa in the phylum Actinobacteria was significantly lower in teak-planting soils than in the native forest soils (compared at a specific planting location). The study also confirmed that the teak’s most common fungal symbionts are arbuscular mycorrhizal fungi, not ectomycorrhizal ones. 

Actinobacteria are Gram-positive bacteria and are notably responsible for producing diverse bioactive compounds, including antimicrobial agents. Hence, their principal plant growth-promoting role is to protect their commensal plants from pathogenic microbes. Only a few studies reported on actinobacteria that live in the teak rhizosphere [18,19]. The impacts of actinobacteria and their synergistic interactions with other rhizosphere microbes in enhancing teak growth and development still need further investigation. For arbuscular mycorrhizal fungi of teak, some previous studies revealed that over 85 species of the phylum Glomeromycota dominated teak rhizosphere, while *Glomus* and *Acaulospora *were the predominant genera [20]. Consistently, a survey study in Thailand also supported that many Glomeromycota species were indigenous teak root symbionts [21]. Some studies proved that optimized teak growth and development highly rely on arbuscular mycorrhizal fungi that enhance soil nutrient and mineral uptake of teak [17,22,23]. Here, we aimed to search for beneficial teak rhizosphere/root endophytic microbes and assess their capability to promote teak growth and development in the nursery stage. Either individual or consortium applications of the selected microbes were tested. The impact of teak rhizobacteria (particularly for actinobacteria) on teak rhizosphere functions and their synergistic interactions with selected teak arbuscular mycorrhizal fungi were focally discussed.

## 2. Materials and Methods

### 2.1. Plant Growth-Promoting Microbes and Their Sources 

An endophytic diazotrophic bacterium isolate CGC-5 was isolated from teak roots collected from Chiang Mai, Thailand (Table 1). Root samples were kept in a cooler during the delivery to the laboratory. All roots were washed and cleaned with running tap water and randomly cut into a piece with 1 cm length. These prepared roots (1 g) were surface sterilized following the protocol described by Rangjaroen et al. [24]. Briefly, teak root segments were soaked in a series of 70% *v*/*v* ethyl alcohol for 1 min, 2% *v*/*v* NaOCl for 2 min, 95% *v*/*v* ethyl alcohol for 30 s, and 30% *v*/*v* H_2_O_2_ for 1 min. Surface-sterilized roots were ground in the presence of 1.5 mL sterile saline (0.85% *w*/*v*) using sterile mortar and pestle. The supernatant (1 mL) of the ground plant material was serially 10-fold diluted, and 0.1 mL of each dilution (10^−1^ to 10^−4^ dilution factors) was spread on the Burk’s agar medium (a nitrogen-free medium) [25]. Seeded Burk’s agar plates were incubated at 30 °C for seven days. The bacterial colony presented on the agar medium was collected and sub-cultured on the same medium until becoming an axenic culture. The capability to fix atmospheric nitrogen of isolate CGC-5 was estimated following a modified acetylene reduction assay addressed by Wiriya et al. [26], and its nitrogenase activity was 10.57 ± 0.26 nmol h^−1^ per mg cell protein. The isolate was preserved in 20% (*v*/*v*) glycerol at −20 °C before further investigation.

A rhizosphere actinobacterium isolate TCM1-050 was isolated from the teak rhizosphere soil collected from the tropical teak plantation in the Forest Industrial Organization, Chiang Mai, Thailand (Table 1). Rhizosphere soil samples were randomly taken near the tree trunk with 30 cm depth from the ground (closed to the root system), kept in a plastic bag, and preserved at 4 °C before delivery to the laboratory. The soil samples were air-dried at an ambient temperature (25 ± 2 °C) for one week, then pretreatment with moist heating. Briefly, 1 g of air-dried soil was resuspended in 9 mL sterile distilled water, mixed well, and incubated at 50 °C for 10 min. The soil supernatant was serially 10-fold diluted, and 0.1 mL of each dilution (10^−3^ to 10^−4^ dilution factors) was spread on the actinomycete isolation agar medium (Difco^TM^, Wayne, PA, USA) and incubated at 30 °C for one month. The actinobacterial colony that appeared on the agar medium was collected and sub-cultured on the International *Streptomyces* Project Medium 2 (ISP2) [27] until becoming an axenic culture. Isolate TCM1-050 was preserved in the same way as for isolate CGC-5. 

An arbuscular mycorrhizal fungus, *Claroideoglomus* sp. PBT03 was supplied by the Research Center of Microbial Diversity and Sustainable Utilization Laboratory, Faculty of Science, Chiang Mai University, Thailand. Isolate PBT03 was identified and classified based on morphological characteristics of its mycelia, arbuscules, and spores into the genus *Claroideoglomus*. Spores of this fungus were collected and maintained by Chaiyasen et al. [28]. Before use in this study, spore viability, fertility, and number were checked and propagated using a pot culture method under greenhouse conditions, in which sweet corn served as a host plant, and the planting substrate was a mixture of sterile sand and soil (1:1 *v*/*v*). Sterilization of the planting substrate was conducted by autoclaving twice at 121 °C for 30 min with a day interval. After planting for three months, plant roots and the whole substrate were collected and kept in a plastic bag at 4 °C. The spore density in the substrate was assessed by counting under a stereomicroscope (Olympus SZ-ST, Tokyo, Japan) after wet sieving and 50% sucrose centrifugation, described elsewhere in Chaiyasen et al. [28]. This spore-containing substrate was used further as the fungal inoculum in pot experiments. 

### 2.2. Identification and Classification of Teak Rhizobacteria

Teak rhizobacteria, isolates CGC-5 and TCM1-050, were identified and classified using some morphological and genotypic data. Isolate CGC-5 was regrown from its glycerol stock on the Burk’s nitrogen-free agar medium and incubated at 30 °C for seven days, while isolate TCM1-050 was regrown on a set of International *Streptomyces* Project Media [29] and incubated at 30 °C for 14 days. The colony morphology and the Gram stain of both isolates were determined and recorded. The biomass of each rhizobacterium was collected and used for molecular identification targeting the 16S rRNA gene. The DNA was extracted from the biomass using FavorPrep^TM^ Tissue Genomic DNA extraction Mini Sample Kit (FAVORGEN^®^, Ping-Tung, Taiwan), following the manufacturer’s instructions. 

The target gene was amplified by polymerase chain reaction (PCR) conducted in a thermocycler (MJ Mini^TM^ BIO-RAD, Foster City, CA, USA). A set of primers 27F (5′-AGAGTTTGATCCTGGCTCAG-3′) and 1492R (5′-GGTTACCTTGTTACGACTT-3′) [30] were used in the gene amplification. PCR reactions (20 µL) consisted of 10 µL DNA polymerase (i-Tag^TM^, iNtRON, Gyeonggi-do, Korea), 6 µL sterile deionized water, 2 µL template DNA, and 1 µL of each primer. The thermal program comprised of an initial denaturation at 95 °C for 10 min, 30 cycles of denaturation at 95 °C for 30 s, annealing at 55 °C for 30 s, and extension at 72 °C for 60 s, and a final extension at 72 °C for 10 min. PCR products were confirmed with agarose gel electrophoresis and purified using NucleoSpin^®^ Gel and PCR Clean-up Kit (MACHEREY-NAGEL GmbH & Co. KG, Düren, Germany), following the manufacturer’s instructions. 

The 16S rRNA gene sequences were retrieved from purified PCR products using an external sequencing service provided by 1st Base Laboratories, Malaysia. Two primers [27F and 1492R] and six primers [27F, 1492R, 530F (5′-GTGCCAGCMGCCGCGG-3′), 907R (5′-CCGTCAATTCMTTTRAGTTT-3′), 1110F (5′-GCAACGAGCGCAACCC-3′), and 520R (5′-ACCGCGGCKGCTGGC-3′) [31,32] were used in the sequencing of isolates CGC-5 and TCM1-050, respectively. The gene sequences obtained were edited and configured as a contiguous consensus by CodonCode [33]. The completed gene sequences were compared to other publicly available gene sequences in EzBioCloud (https://www.ezbiocloud.net/, accessed on 7 August 2021) and GenBank–NCBI BLAST^®^ (https://blast.ncbi.nlm.nih.gov/Blast.cgi, accessed on 7 August 2021) databases. Multiple gene sequences showing a high percentage (>98.5%) of the gene sequence similarity were collected from both databases and aligned with the MUSCLE algorithm in MEGA X software (https://www.megasoftware.net, accessed on 7 August 2021), where a variety of phylogenetic trees were analyzed and constructed.

### 2.3. In Vitro Plant Growth-Promoting Trait Assessments of Teak Rhizobacteria

Auxins are a class of phytohormones, playing a crucial role in plant growth and development. The auxin family consists of diverse molecular members such as IAA (the most abundant and basic auxin), indole-3-butyric acid, indole-3-propionic acid, etc. The capability to form auxins was estimated using a modified Salkowski’s colorimetric method, described in Wiriya et al. [26]. Briefly, rhizobacteria were grown in 5 mL liquid media (Burk’s broth for isolate CGC-5 and ISP2 broth for isolate TCM1-050). The media were supplemented with 2% (*w*/*v*) L-tryptophan (Sigma-Aldrich^TM^, Shanghai, China). Every culture broth was incubated in the dark on a reciprocal shaker (110 rpm) at an ambient temperature (25 ± 2 °C) for three (isolate CGC-5) and seven (isolate TCM1-050) days. Then, the incubated culture broth was centrifuged at 6693× *g* for 5 min. Then, 1 mL of the supernatant was transferred and mixed with 2 mL of Salkowski’s reagent (1 mL of 0.5 M FeCl_3_ in 50 mL of 35% HClO_4_), followed by incubation in the dark for 30 min. The mixture developed a pink-to-red color, indicating the production of an indole acetic acid (auxins)-like molecule from L-tryptophan. The concentration (in µg mL^−1^) of auxins corresponded to the absorbance at 530 nm wavelength, measured by a spectrophotometer (Thermo Scientific GENESYS™ 20, Waltham, MA, USA) and compared with a standard curve made of the known concentrations (5–100 µg mL^−1^) of L-tryptophan. 

Ammonia (NH_3_) as a mother source of the ammonium ion (NH_4_^+^) is a major source of nitrogen in the plant rhizosphere and essential for plant growth and development. Hence, the capability to produce ammonia is a vital plant growth-promoting trait of beneficial rhizosphere microbes. Rhizobacteria were grown in 5 mL peptone (Difco^TM^, USA) water at 27 °C on a reciprocal shaker (110 rpm) for ten days to test their ammonia-forming ability [34]. The culture broth was centrifuged at 6693× *g* for 5 min, and the supernatant was mixed with an equal volume of Nessler’s reagent [35] and incubated for 3–5 min. The mixture developed a deep yellow-to-brown color, signifying positive ammonia production. 

Environmental stress-induced reactive oxygen species (ROS) generation ultimately hampers plant growth and development. Catalase is an antioxidant enzyme that protects living cells from oxidative damage caused by ROS. The ability to produce this enzyme by rhizobacteria was tested by dropping the 3% (*v*/*v*) hydrogen peroxide (Sigma-Aldrich^TM^, China) solution on their culture or colony. Bubbles formed after dropping the solution indicate positive catalase production.

### 2.4. Pot-Scale Plant-Growth-Promoting Activity Assessments of Teak Rhizosphere Microbes

**Figure 1:**
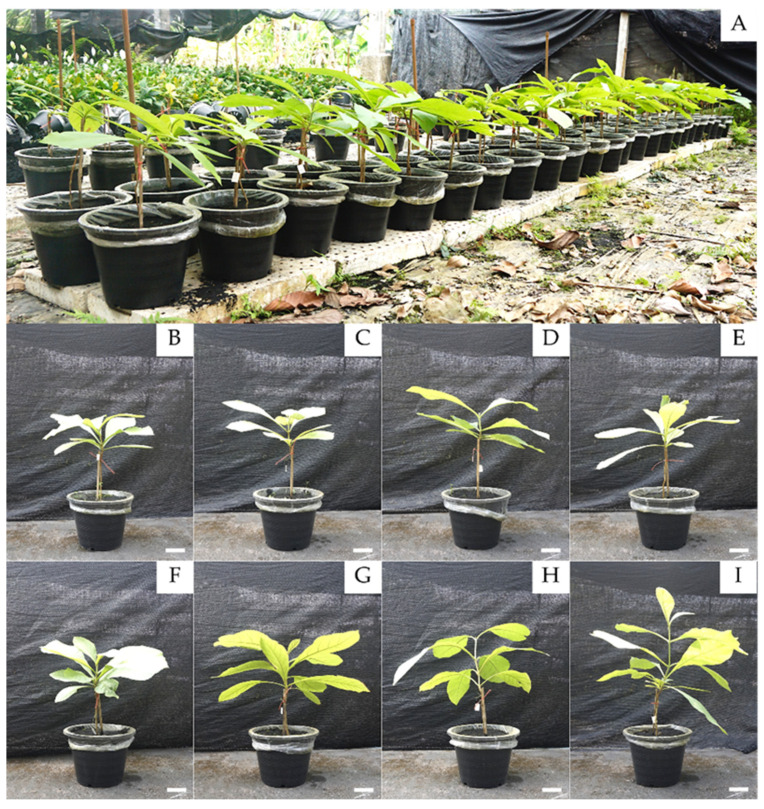
Establishment of pot experiments for assessing plant-growth-promoting activity of teak rhizosphere microbes. Pot experiments were conducted under greenhouse conditions (**A**). A comparison of representative plant physiology derived from different treatments at the end of pot experiments (**B**–**I**). The treatments include teak seedlings grown in planting materials non-inoculated (control, **B**) and inoculated with different rhizosphere microbes, i.e., with nitrogen-fixing bacterium isolate CGC-5 (**C**), with actinobacterium isolate TCM1-050 (**D**), with isolates CGC-5 and TCM1-050 (**E**), with arbuscular mycorrhizal fungus, *Claroideoglomus *isolate PBT03 (**F**), with isolates CGC-5 and PBT03 (**G**), with isolates TCM1-050 and PBT03 (**H**), and with every tested microbial isolate (**I**). Scale bars = 7 cm.

Pot experiments (Figure 1) were conducted to determine plant-growth-promoting activity of teak rhizosphere microbes (either individuals or in consortia) under greenhouse conditions (35 ± 2 °C in daytime, 12 h light-dark cycle, and 56 ± 5% humidity) for three months (January–April 2019 in Chiang Mai, Thailand) with a completely randomized design (five replicates per each treatment). The treatments comprised of (1) control (no microbial inoculum added); (2) CGC-5 (inoculated with isolate CGC-5); (3) TCM1-050 (inoculated with isolate TCM1-050); (4) CGC-5 + TCM1-050 (inoculated with isolates CGC-5 and TCM1-050); (5) PBT03 (inoculated with isolate PBT03); (6) CGC-5 + PBT03 (inoculated with isolates CGC-5 and PBT03); (7) TCM1-050 + PBT03 (inoculated with isolates TCM1-050 and PBT03); and (8) CGC-5 + TCM1-050 + PBT03 (inoculated with every tested microbe). 

The nitrogen-fixing bacterium isolate CGC-5 was grown in nutrient broth (Difco^TM^, USA) on a reciprocal shaker (120 rpm) at 27 °C for two days. Its cells were collected by centrifugation at 6693× *g* for 5 min and resuspended in sterile distilled water. The cell density was adjusted to 10^8^ colony forming units (CFUs) mL^−1^ using sterile distilled water and counting with a hemocytometer (HBG, Giessen, Germany) under a light microscope (Olympus CH30, Tokyo, Japan). The actinobacterium isolate TCM1-050 was grown on ISP3 agar medium [29] at 30 °C for 14 days. Its cells and spores were collected by flooding the seeded agar plate with sterile distilled water containing tween 80 (a drop of tween 80 per 500 mL water) and adjusted to 10^6^ spores mL^−1^ using the hemocytometer-based method mentioned above. For the arbuscular mycorrhizal fungus, spores of *Claroideoglomus* sp. PBT03 were propagated and quantified as described previously. A spore density at 50 fungal spores per g of planting materials was measured and prepared as the fungal inoculum. 

Teak seedlings (3-months-old) prepared by a micropropagation approach were supplied by Thai Orchid Labs Co., Ltd., Bangkok, Thailand. Only healthy and uniform teak seedlings were selected from the nursery stocks. Rice husk charcoal mixed with coconut husk (1:1 *v*/*v*) served as the planting material. These planting substrates have plenty of pores, which is good for microbial establishment. Each teak seedling was transplanted into a plastic pot filled with 5 L of the planting material. Then, 20 mL of rhizobacterial inoculum or 20 g of the fungal inoculum (~1000 spores) was applied at the seedling rhizosphere of any microbial treatments. Substrate moisture and compositions in every treatment were balanced by adding equal volumes of sterile distilled water used in the bacterial inoculum preparation (with or without tween 80) and/or the planting material used in the fungal inoculum preparation [sand and soil (1:1 (*v*/*v*)]. 

Plant growth and development indexes, including stem length, leaf number per plant, stem circumference, fresh and dry weights of stem and root, and leaf phytochemical compositions (i.e., chlorophyll content, nitrogen, and phosphorus), were assessed and recorded to determine plant growth-promoting potentials of teak rhizosphere microbes tested in each treatment. Stem length and leaf number per plant were measured every month until the end of the experiments, while the other indexes were detected only at the end of the experiments. Dry weights of stems and roots were evaluated after drying plant materials in a hot air oven at 60 °C for 72 h. Leaf chlorophyll concentration was estimated after extracting 5 g of fresh leaves with 80% ethanol, following a protocol described by Jiang et al. [36]. For leaf nitrogen and phosphorus contents, teak leaves were collected and dried at 60 °C for 72 h before measuring with a protocol described by Zhang et al. [37].

Plant root colonization by arbuscular mycorrhizal fungi is beneficial for ensuring a long-term synergistic interaction between host plants and their fungal partners. The capability of arbuscular mycorrhizal fungi to colonize teak roots was determined by the percentage of root colonization and the number of spores produced in the rhizosphere soil, described by Chaiyasen et al. [28] and Wiriya et al. [26]. At the end of pot experiments, planting materials and plant roots were collected from each pot. For the percentage of root colonization, root samples were washed with running tap water and cut into small pieces (~1 cm in length). Root tissue pigments were removed using 10% potassium hypochlorite (Union Science, Chiang Mai, Thailand) and further stained with 0.05% trypan blue (Sigma-Aldrich^TM^, China). Stained root segments were randomly selected, placed on a glass slide, mounted with polyvinyl alcohol-lactic acid-glycerol solution (each composition purchased from Sigma-Aldrich^TM^, China–for each chemical), and observed under a light microscope. The percentage of root colonization was calculated using the formula: (number of root segments showing colonization/total number of root segments observed) × 100. For the number of fungal spores produced in the teak rhizosphere, all planting materials derived from the pot were dried at an ambient temperature (25 ± 2 °C) for one day before counting. Fungal spores in 100 g of the dried planting materials were isolated and quantified by wet sieving and the sucrose centrifugation method as described previously.

### 2.5. Statistical Analysis 

The derived results were reported as means ± standard deviations (SDs). The statistical comparison of means was conducted with one-way analysis of variance (one-way ANOVA) with Tukey’s post hoc test in SPSS Statistics software version 26 (IBM Corporation, Somers, NY, USA). The statistical values tested at *α* = 0.05 were reported with *F*-distribution, degrees of freedom, and significant level (*p*).

## 3. Results

### 3.1. Teak Rhizobacteria and Their Plant Growth-Promoting Traits

**Figure 2:**
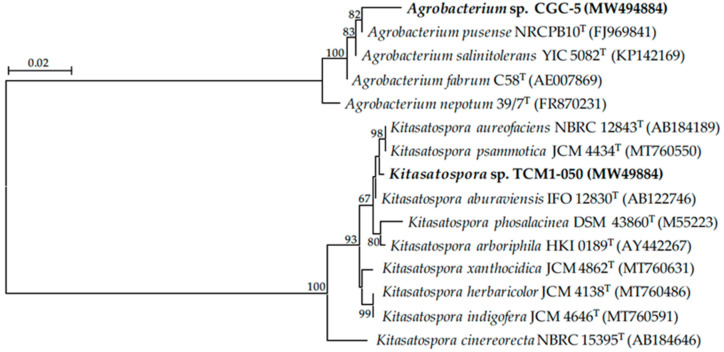
Unrooted neighbor-joining phylogenetic tree of teak rhizobacteria in this study. The tree was constructed using 16S rRNA gene sequence data derived from isolates CGC-5 and TCM1-050 (in bold) and their closely related phylogenetic species. The GenBank accession number of the gene sequence is presented in the parenthesis. Bootstrap values (based on 1000 replications) of >60% are at the tree’s nodes, and the scale bar represents 2% dissimilarity.

Based on morphological and genotypic characteristics of teak rhizobacteria used in this study (Table 1), they belong to the genera *Agrobacterium* and *Kitasatospora*. Isolate CGC-5 is a Gram-negative bacterium that can produce indole acetic acid-like molecules (106.29 ± 2.20 µg mL^−1^) and catalase, but not ammonia (Table 1). Its 16S rRNA gene sequence data and phylogenetic analysis (Table 1 and Figure 2, Figure S1 and Figure S2) suggested that isolate CGC-5 was closely related to *Agrobacterium pusense* NRCPB10^T^ and *Agrobacterium salinitolerans* YIC 5082^T^ supported by the gene sequence similarities of 99.02% (determined by NCBI BLAST^®^).

Isolate TCM1-050 is a Gram-positive bacterium that can produce indole acetic acid-like molecules (163.86 ± 1.01 µg mL^−1^), ammonia, and catalase (Table 1). When comparing the 16S rRNA gene sequence data of this isolate using the EzBioCloud database, it was closely related to *Kitasatospora aureofaciens* NBRC 12843^T^ supported by the gene sequence similarity of 99.63% (Table 1). However, its closest species was *Kitasatospora psammotica* JCM 4434^T^ (98.52% gene sequence similarity) when using the GenBank–NCBI BLAST^®^ database. The phylogenetic analyses also revealed that isolate TCM1-050 fell in the same clade with *K*. *aureofaciens* and *K*. *psammotica* (Figure 2, Figure S1 and Figure S2). 

### 3.2. Plant-Growth-Promoting Activity of Teak Rhizosphere Microbes in Pot-Scale Assessments

**Figure 3:**
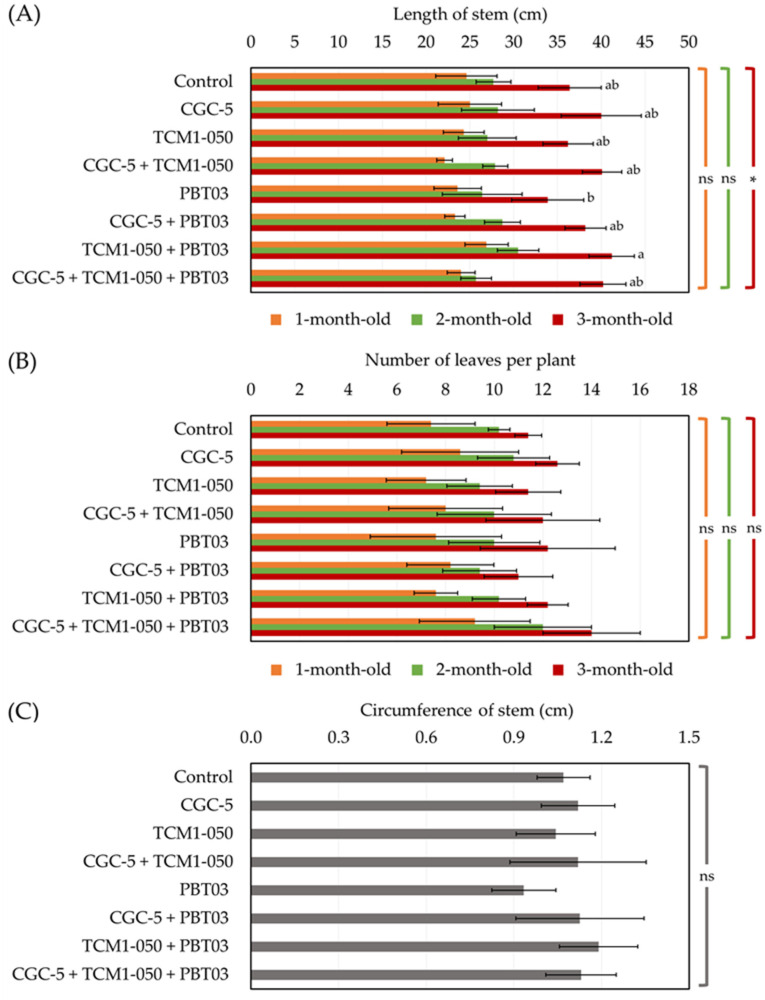
Impacts of teak rhizosphere microbes on stem length (**A**), leaf number per plant (**B**), and stem circumference of teak seedlings. Different treatments were established, including an uninoculated control and those inoculated with *Agrobacterium* sp. CGC-5, *Kitasatospora* sp. TCM1-050, *Claroideoglomus* sp. PBT03, or different consortia of these microbes. The experiments were done in five replicates. Small case letters refer to the statistical difference of means compared by one-way ANOVA at *α* = 0.05. The statistical results comprise of (**A**) length of stem at 1-months-old (*F*_(7, 32)_ = 1.60, *p* = 0.17), 2-months-old (*F*_(7, 32)_ = 1.29, *p* = 0.29), and 3-months-old (*F*_(7, 32)_ = 3.13, *p* ≤ 0.0005), (**B**) number of leave per plant at 1-months-old (*F*_(7, 32)_ = 0.53, *p* = 0.80), 2-months-old (*F*_(7, 32)_ = 1.36, *p* = 0.25), and 3-months-old (*F*_(7, 32)_ = 1.52, *p* = 0.195), and (**C**) circumference of stem (*F*_(7, 32)_ = 1.25, *p* = 0.31). The asterisk remark indicates the statistical significance, while “ns” refers to “no significance”.

Based on pot experiments conducted to assess the plant-growth-promoting potentials of teak rhizosphere microbes, either applying individually or in forms of consortia could support teak growth and development in different ways (no death plant was found for the entire period of pot experiments). No microbes affected the length of teak seedling stems when measured after transplanting for up to two months (Figure 3A). A significant difference in stem lengths was observed at the end of the experiments (Figure 1 and Figure 3A). The shortest stem length was found in the treatment inoculated with the arbuscular mycorrhizal fungus (*Claroideoglomus* sp. PBT03) only. The longest stem length was detected in the treatment inoculated with the actinobacterium (*Kitasatospora* sp. TCM1-050) and *Claroideoglomus* sp. PBT03 (Figure 3A). During the first two months of pot experiments, teak stem lengths increased approximately 3.5 cm, but the length increased rapidly over 10 cm one month later (Figure 3A). For the number of leaves per plant (Figure 1 and Figure 3B) and the stem circumference (Figure 1 and Figure 3C), no significant difference in these indexes was observed between any treatments. At the end of the experiment, there were approximately 12 leaves per plant in any treatments, and the highest leaf count (14 leaves per plant) was found in the treatment inoculated with every tested microbe (Figure 1 and Figure 3B). The longest stem circumference (1.16 cm) was measured in the treatment inoculated with *Kitasatospora* sp. TCM1-050 and *Claroideoglomus* sp. PBT03, while the shortest one (0.93 cm) was detected in the treatment inoculated solely with *Claroideoglomus* sp. PBT03 (Figure 1 and Figure 3C).

**Figure 4:**
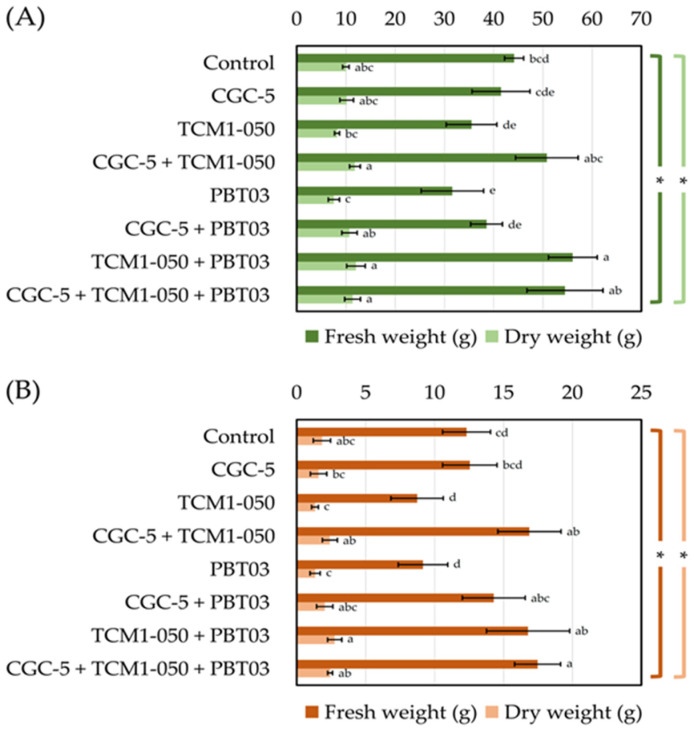
Impacts of teak rhizosphere microbes on fresh and dry weights of teak seedlings’ stems (**A)** and roots (**B**). Different treatments were established, including a non-inoculated control and those inoculated with *Agrobacterium* sp. CGC-5, *Kitasatospora* sp. TCM1-050, *Claroideoglomus* sp. PBT03, or different consortia of these microbes. The experiments were done in five replicates. Small case letters refer to the statistical difference of means compared by one-way ANOVA at *α* = 0.05. The statistical results comprise of (**A**) fresh (*F*_(7, 32)_ = 13.40, *p* ≤ 0.0005) and dry (*F*_(7, 32)_ = 7.83, *p* ≤ 0.0005) weights of teak stems and (**B**) fresh (*F*_(7, 32)_ = 13.05, *p* ≤ 0.0005) and dry (*F*_(7, 32)_ = 6.00, *p* ≤ 0.0005) weights of teak roots. The asterisk remark indicates the statistical significance.

Teak rhizosphere microbes significantly affected the fresh and dry weights of teak seedling shoots and roots (Figure 4). The highest fresh weights of teak stems were measured in treatments inoculated with *Kitasatospora* sp. TCM1-050 + *Claroideoglomus* sp. PBT03, with every tested microbe, and with *Agrobacterium* sp. CGC-5 + *Kitasatospora* sp. TCM1-050 (Figure 4A). These results were consistent with those observed for the dry weight of teak stems. The lowest fresh and dry weights of teak stems were found in the treatment inoculated solely with *Claroideoglomus* sp. PBT03 (Figure 4A). Similar results were observed with teak roots’ fresh and dry weights, although the treatment inoculated solely with *Kitasatospora* sp. TCM1-050 also showed the lowest results (Figure 4B).

**Figure 5:**
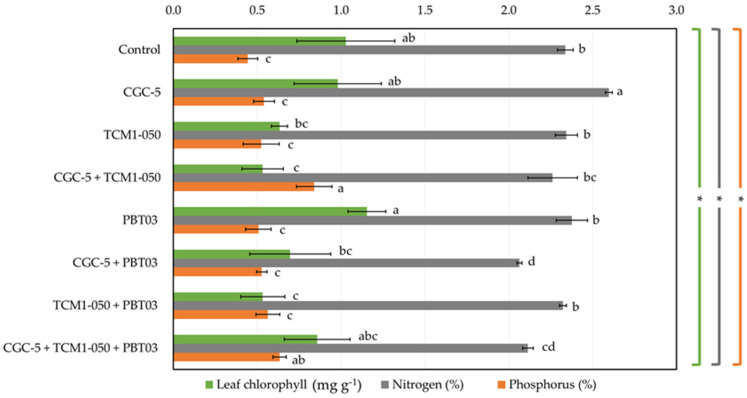
Impacts of teak rhizosphere microbes on teak leaf phytochemicals. Different treatments were established, including a non-inoculated control and those inoculated with *Agrobacterium* sp. CGC-5, *Kitasatospora* sp. TCM1-050, *Claroideoglomus* sp. PBT03, or different consortia of these microbes. The experiments were done in five replicates. Small case letters refer to the statistical difference of means compared by one-way ANOVA at *α* = 0.05. The statistical results comprise of chlorophyll (*F*_(7, 32)_ = 7.59, *p* ≤ 0.0005), nitrogen (*F*_(7, 32)_ = 16.57, *p* ≤ 0.0005), and phosphorus (*F*_(7, 32)_ = 7.98, *p* ≤ 0.0005) contents of teak leaves. The asterisk remark indicates the statistical significance.

Some teak leaf phytochemical contents varied depending upon different treatments tested (Figure 5). The average chlorophyll content was 0.80 mg g^−1^, while its highest value (1.15 mg g^−1^) was found in the treatment inoculated solely with *Claroideoglomus* sp. PBT03 (Figure 5). Any treatments inoculated with *Kitasatospora* sp. TCM1-050 showed relatively low chlorophyll content. The percentage of leaf nitrogen content ranged from 2.06% to 2.60% (Figure 5), and its highest value was observed in the treatment inoculated only with *Agrobacterium* sp. CGC-5. When *Agrobacterium* sp. CGC-5 and *Claroideoglomus* sp. PBT03 were co-inoculated, the lowest leaf nitrogen content was detected. Many microbial inocula did not change the leaf phosphorus content compared to the non-inoculated control (Figure 5). The average leaf phosphorus content was 0.57%, while the highest value (0.84%) was found in the treatment with *Agrobacterium* sp. CGC-5 and *Kitasatospora* sp. TCM1-050, followed by the medium value (0.59%) detected in the treatment with every microbial inoculum.

**Figure 6:**
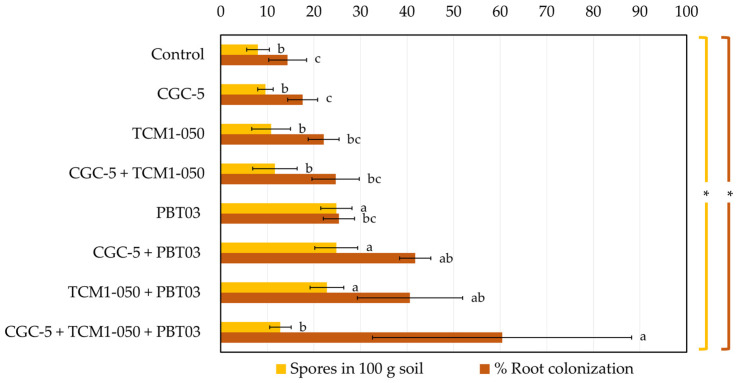
Spore counts and root colonization percentages of arbuscular mycorrhizal fungi. Different treatments were established, including a non-inoculated control and those inoculated with *Agrobacterium* sp. CGC-5, *Kitasatospora* sp. TCM1-050, *Claroideoglomus* sp. PBT03, or different consortia of these microbes. The experiments were done in five replicates. Small case letters refer to the statistical difference of means compared by one-way ANOVA at α 0.05. The statistical results comprise of arbuscular mycorrhizal spores in 100 g soil (*F*_(7, 32)_ = 20.75, *p* ≤ 0.0005) and root colonization percentages (*F*_(7, 32)_ = 9.71, *p* ≤ 0.0005). The asterisk remark indicates the statistical significance.

**Figure 7:**
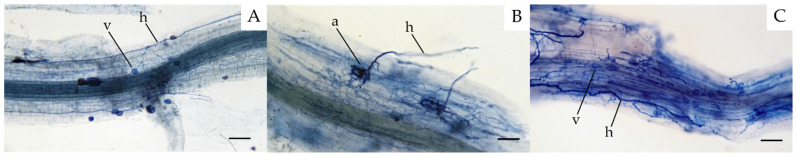
Micrographs of teak roots colonized by arbuscular mycorrhizal fungi. Teak roots were stained with 0.05% trypan blue before micrography under a light microscope, where a = appressorium, h = hypha, and v = vesicle of fungi. Root samples were collected from different treatments in pot experiments, including a non-inoculated control (**A**), the treatment inoculated with *Kitasatospora* sp. TCM1-050 and *Claroideoglomus* sp. PBT03 (**B**), and the treatment inoculated with every tested microbe (**C**). Scale bars = 10 µm.

The capability of the arbuscular mycorrhizal fungus (*Claroideoglomus* sp. PBT03) to colonize plant roots and produce spores in the teak rhizosphere was assessed to ensure a long-term mutual support between the fungus and teak plants (Figure 6 and Figure 7). At the end of the pot experiments, significantly higher numbers of spores were counted in the treatments inoculated with the fungus and the fungus plus each tested teak rhizobacterium (23–25 spores in 100 g soil), compared to the non-inoculated control and other treatments (8–13 spores in 100 g soil). The numbers of fungal spores produced in the presence of each rhizobacterium corelated with the percentages of root colonization by the fungus. However, the highest percentage of root colonization (60.40%) was found in the treatment inoculated with the fungus and both rhizobacteria, in which the spore count was significantly low (13 spores in 100 g soil).

## 4. Discussion

A greater number of studies have explored the community structure of arbuscular mycorrhizal fungi in teak rhizospheres [18,20,21,22,23,38], while only a few studies focused on teak rhizobacteria [18,19]. The unique synergistic interactions between teak trees and arbuscular mycorrhizal fungi are well characterized and recognized so far [4,39,40]. A recent metagenomics study confirmed that teak growth and development were more dependent on the interactions with arbuscular mycorrhizal fungi than those with ectomycorrhizal ones [18]. This unique dependency was also observed in our work and confirmed with the presence of arbuscular mycorrhizal fungi in teak rhizospheres, even in the non-inoculated controls, suggesting that these fungal symbionts form mutual interactions with teak trees since the early stage of tree seedling development. 

With metagenomics evidence, Proteobacteria and Acidobacteria predominated the rhizospheres of teak trees grown as monocultures in the forest soil [18]. Our study is consistent with this report as our isolated teak root endophyte, *Agrobacterium* sp. CGC-5 also belongs to the phylum Proteobacteria (class Alphaproteobacteria). Although most of the genus *Agrobacterium*’s members are associated with plants and play crucial roles in plant growth and development [41], a study reported recently that *Ag*. *tumefaciens* species complex could cause crown gall disease in teak nursery clones [9]. Based on our investigation, *Agrobacterium* sp. CGC-5 did not show any apparent disease symptoms in teak seedlings. Moreover, it exhibited distinguished teak-growth-promoting activities even when applied individually, supported by the highest teak leaf nitrogen content detected in this microbial treatment.

Actinobacteria are notable producers of antimicrobial agents, which is a key feature that can be applied as living defenders to protect their plant hosts from pathogens. To our knowledge, teak-growth-promoting roles of actinobacteria and how these bacteria interact with other teak rhizosphere microbes are still largely unknown. Only a study by Madhaiyan et al. [19] discovered a novel actinobacterial species, *Leifsonia soli*, from teak rhizosphere soil. Interestingly, another study unveiled that the abundance of the phylum Actinobacteria in the forest soil planted with teak monocultures was relatively lower than that in the same soil grown with diverse native forest species [18]. This decreased dependency between teak trees and actinobacteria might be a result of bioactive compounds produced by both partners. 

Teak is a well-known wooden tree that withstands pest invasion because many anti-pest bioactive compounds are found in its leaves [42,43] and woods [44]. However, the phytochemical compositions of teak root exudates are yet to be profiled to better understand how teak trees shape their rhizosphere microbial community structures. Our study found that teak rhizospheres house plenty of actinobacteria (data not shown) and *Kitasatospora* sp. TCM1-050 showed better plant growth-promoting traits than the others. Isolate TCM1-050 also worked well with *Agrobacterium* sp. CGC-5 in supporting teak phosphorus uptake. Moreover, this teak rhizosphere actinobacteria didn’t inhibit teak root colonization by *Claroideoglomus* sp. PBT03. Therefore, these teak rhizobacteria may act as mycorrhiza helper bacteria [45] to support mycorrhizal functions to promote soil nutrient/mineral uptake by host plants.

Some recent studies demonstrated the utilization of diverse beneficial microbes like plant growth-promoting rhizobacteria and arbuscular mycorrhizal fungi to promote teak growth and development [16,17]. These studies pointed out the importance of teak growth and development at the nursery phase, which is crucial to warrant the long-term productivity of this agroforestry species. Based on our teak-growth-promoting assessments at the nursery stage, either applied teak rhizosphere microbes individually or in consortia, teak growth and development highly relied on tested microbes. Non-inoculation or inoculation with microbes did not significantly alter the teak seedlings’ stem length and circumference. This insignificant difference in teak morphology might be a result of experimental duration. Our observation was conducted every month for up to 3 months, which might be too short to discriminate the impacts of microbial inoculants. Raghu et al. [16] found that a microbial consortium (made of *Am*. *leptoticha*, *Az*. *chroococcum*, and *Trichoderma harzianum*) could significantly increase the teak seedlings’ stem length and circumference observed on day 180th of their experiments.

It was apparent that individual microbes often showed lower promoting effects on teak growth and development than those inoculated in the form of a consortia. This result was supported by the significantly low biomass (fresh and dry weights) of teak stems and roots, especially when teak seedlings were inoculated with *Kitasatospora* sp. TCM1-050 or *Claroideoglomus* sp. PBT03. When these two microbes were applied together, teak seedlings could produce the maximum volumes of stem and root biomass. Although arbuscular mycorrhizal fungi are mainly responsible for the soil mineral/nutrient uptake of their host plants, it is evident that they require support/interactions with other rhizosphere microbes [45]. This fact was also confirmed in our study using the percentages of teak root colonization by *Claroideoglomus* sp. PBT03, which were greater in the presence of teak rhizobacteria compared to the other treatments and non-inoculated control. 

A similar observation was reported when a plant growth-promoting rhizobacterium (*B*. *subtilis*) and an arbuscular mycorrhizal fungus (*R*. *clarus*) were tested either individually or in a consortium for their teak-growth-promoting activities [17]. The authors revealed that teak root length and density were significantly increased when both microbes were applied together. These findings also showed a positive correlation with teak root absorption of soil minerals. Another long-term study confirmed that teak seedlings supplemented with a mixture of microbes (*Am*. *leptoticha*, *Az*. *chroococcum*, and *T*. *harzianum*) could sustain teak growth and enhance teaks’ biovolume up to 289% greater than non-inoculated plants (assessed 73 months after out-planting) [16]. 

## 5. Conclusions

Teak growth and development were highly dependent on the interactions with its beneficial rhizosphere microbes. In this study, teak rhizosphere/root-associated microbes, including a nitrogen-fixing bacterium, *Agrobacterium* sp. CGC-5, an actinobacterium, *Kitasatospora* sp. TCM1-050, and an arbuscular mycorrhizal fungus, *Claroideoglomus* sp. PBT03 were isolated and tested for their teak-growth-promoting activities at the nursery stage. Our findings suggested that applying these microbes in the form of consortia could optimally promote teak growth and development compared to non-inoculated controls and other individual treatments. Besides, the teak-growth-promoting roles of the arbuscular mycorrhizal fungus highly relied on the interactions with other teak rhizobacteria. Additional long-term studies to assess the impacts of the large-scale planting system, seasonal conditions, and forest biodiversity (a variety of forest tree species and forest soil microbiota) on the synergistic interactions between teak trees and these selected microbes are still needed. To this end, this study convinced us that these beneficial teak rhizosphere microbes could be developed further as a biofertilizer to improve teak health during nursery development. The utilization of these beneficial microbes could be a green strategy to promote sustainable teak agroforestry in the future.

## Figures and Tables

**Table 1:** Teak-derived rhizobacteria used in this study.

Source Information and Characteristics	Isolate CGC-5	Isolate TCM1-050
Geographical location (latitude–longitude)	Muang Chiang Mai District, Chiang Mai, Thailand (18°50′15.6″ N, 98°57′58.0″ E)	Mae Chaem District, Chiang Mai, Thailand (18°18′18.7″ N, 98°21′47.3″ E)
Age of host plant	~25 years	~15 years
Date of isolation	November 2014	November 2014
Colony color *^a^*	White	Ocher brown
Cell/colony morphology	Short rod cells	Aerial mycelium-forming colony with ocher brown soluble pigment
Gram staining	Gram-negative	Gram-positive
Closest GenBank species (% identity of the 16S rRNA gene sequence)	*Agrobacterium pusense* NRCPB10^T^ (99.02%) *Agrobacterium salinitolerans* YIC 5082^T^ (99.02%)	*Kitasatospora aureofaciens* NBRC 12843^T^ (99.63%)
GenBank accession number for the 16S rRNA gene sequence	MW494884	MW494883
Capability to produce auxins (µg mL^−1^) *^b^*	106.29 ± 2.20	163.86 ± 1.01
Capability to produce ammonia	−	+
Capability to produce catalase	+	+

*^a^* Colony colors of isolates CGC-5 and TCM1-050 were observed after growing them respectively on Burk’s agar medium and International *Streptomyces* Project 2 agar medium. *^b^* Capability to produce auxins was estimated with a colorimetric method and compared to a standard curve made of the known concentrations of L-tryptophan. (−) = not produced and (+) = produced.

## Data Availability

Not applicable.

## Supplementary Materials

The following are available online at https://www.mdpi.com/article/10.3390/microorganisms9091990/s1, Figure S1: Unrooted maximum-likelihood phylogenetic tree of teak rhizobacteria used in this study, Figure S2: Unrooted maximum-parsimony phylogenetic tree of teak rhizobacteria used in this study.
