# Supplementary material for: Enhancing Teak (Tectona grandis) Seedling Growth by Rhizosphere Microbes: A Sustainable Way to Optimize Agroforestry
Source: Microorganisms. 2021 Sep 19;9(9):1990. doi: 10.3390/microorganisms9091990 (PMC8465541; doi:10.3390/microorganisms9091990)

microorganisms

MDPI

Supplementary Materials

Enhancing Teak (*Tectona grandis*) Seedling Growth by Rhizosphere Microbes: A Sustainable Way to Optimize Agroforestry

Leardwiriyakool Chaiya [1], Paiboolya Gavinlertvatana [2], Neung Teaumroong [3], Wasu Pathom-aree [1,4], Amornrat Chaiyasen [5], Rungroch Sungthong [6,*] and Saisamorn Lumyong [1,4,7,*]

[1] Department of Biology, Faculty of Science, Chiang Mai University, Chiang Mai 50200, Thailand; Leardkool@gmail.com (L.C.); wasu.p@cmu.ac.th (W.P.); scboi009@gmail.com (S.L.)
[2] Thai Orchid Labs Co. Ltd., Khannayao, Bangkok 10230, Thailand; thaiorchidslab@gmail.com
[3] School of Biotechnology, Institute of Agricultural Technology, Suranaree University of Technology, Nakhon Ratchasima 30000, Thailand; neung@sut.ac.th
[4] Center of Excellence in Microbial Diversity and Sustainable Utilization, Faculty of Science, Chiang Mai University, Chiang Mai 50200, Thailand; wasu.p@cmu.ac.th (W.P.); scboi009@gmail.com (S.L.)
[5] Soil Science Research Group, Agricultural Production Science Research and Development Division, Department of Agriculture, Ministry of Agriculture and Cooperatives, Bangkok 10900, Thailand; amornrat057@gmail.com
[6] Laboratory of Hydrology and Geochemistry of Strasbourg, University of Strasbourg, UMR 7517 CNRS/EOST, Strasbourg CEDEX 67084, France; sungthong@unistra.fr
[7] Academy of Science, The Royal Society of Thailand, Bangkok 10300, Thailand; scboi009@gmail.com

* Correspondence: sungthong@unistra.fr (R.S.), scboi009@gmail.com (S.L.)

Figure S1. Unrooted maximum-likelihood phylogenetic tree of teak rhizobacteria used in this study. The tree was constructed using 16S rRNA gene sequence data derived from isolates CGC-5 and TCM1-050 (in bold) and their closely related phylogenetic species. The GenBank accession number of the gene sequence is presented in the parenthesis. Bootstrap values (based on 1,000 replications) of >60% are at the tree's nodes, and scale bar represents 2% dissimilarity.

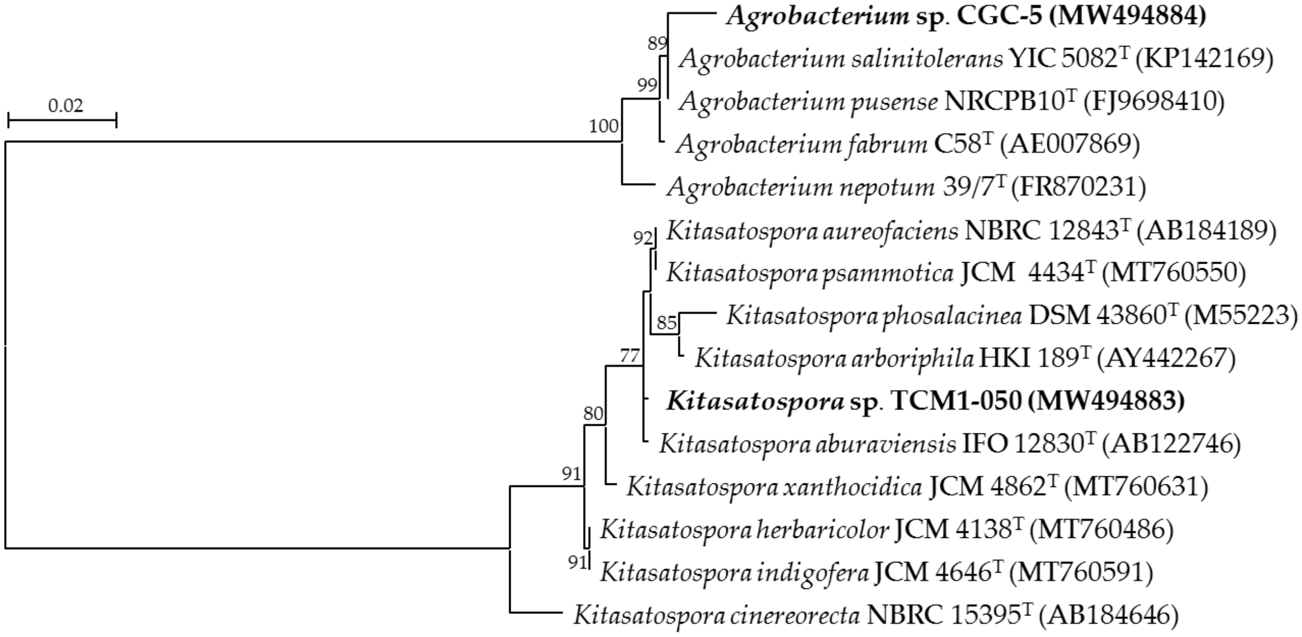

Figure S2. Unrooted maximum-parsimony phylogenetic tree of teak rhizobacteria used in this study. The tree was constructed using 16S rRNA gene sequence data derived from isolates CGC-5 and TCM1-050 (in bold) and their closely related phylogenetic species. The GenBank accession number of the gene sequence is presented in the parenthesis. Bootstrap values (based on 1,000 replications) of >60% are at the tree's nodes.

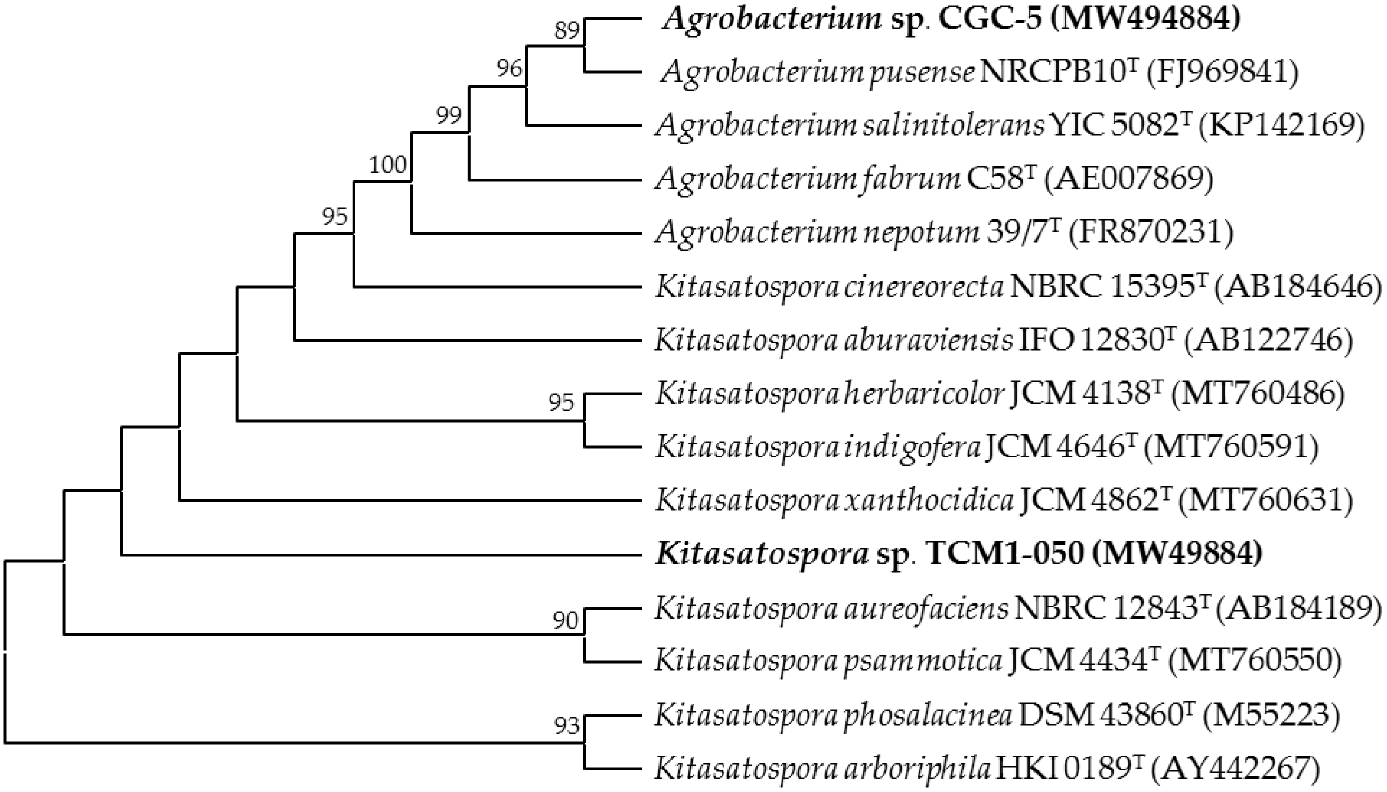

Supplement: Supplementary file 1 [file microorganisms-09-01990-s001.zip › microorganisms-1385314-supplementary.pdf]
